# Diabetic HDL Is Dysfunctional in Stimulating Endothelial Cell Migration and Proliferation Due to Down Regulation of SR-BI Expression

**DOI:** 10.1371/journal.pone.0048530

**Published:** 2012-11-02

**Authors:** Bing Pan, Yijing Ma, Hui Ren, Yubin He, Yongyu Wang, Xiaofeng Lv, Donghui Liu, Liang Ji, Baoqi Yu, Yuhui Wang, Y. Eugene Chen, Subramaniam Pennathur, Jonathan D. Smith, George Liu, Lemin Zheng

**Affiliations:** 1 The Institute of Cardiovascular Sciences, Ministry of Education, Key Laboratory of Cardiovascular Molecular Biology and Regulatory Peptides, Ministry of Health, Peking University Health Science Center, Beijing, China; 2 Institute of Systems Biomedicine, School of Basic Medical Sciences, Ministry of Education, Key Laboratory of Cardiovascular Molecular Biology and Regulatory Peptides, Ministry of Health, Peking University Health Science Center, Beijing, China; 3 Key Laboratory of Molecular Cardiovascular Sciences, Ministry of Education, Key Laboratory of Cardiovascular Molecular Biology and Regulatory Peptides, Ministry of Health, Peking University Health Science Center, Beijing, China; 4 The Military General Hospital of Beijing, Beijing, China; 5 Department of Pathology, Shantou University Medical College, Shantou, Guangdong, China; 6 Department of Medicine, University of Michigan, Ann Arbor, Michigan, United States of America; 7 Department of Cell Biology, Cleveland Clinic, Cleveland, Ohio, United States of America; University of Florida, United States of America

## Abstract

**Background:**

Diabetic HDL had diminished capacity to stimulate endothelial cell (EC) proliferation, migration, and adhesion to extracellular matrix. The mechanism of such dysfunction is poorly understood and we therefore sought to determine the mechanistic features of diabetic HDL dysfunction.

**Methodology/Principal Findings:**

We found that the dysfunction of diabetic HDL on human umbilical vein endothelial cells (HUVECs) was associated with the down regulation of the HDL receptor protein, SR-BI. Akt-phosphorylation in HUVECs was induced in a biphasic manner by normal HDL. While diabetic HDL induced Akt phosphorylation normally after 20 minutes, the phosphorylation observed 24 hours after diabetic HDL treatment was reduced. To determine the role of SR-BI down regulation on diminished EC responses of diabetic HDL, Mouse aortic endothelial cells (MAECs) were isolated from wild type and SR-BI (−/−) mice, and treated with normal and diabetic HDL. The proliferative and migratory effects of normal HDL on wild type MAECs were greatly diminished in SR-BI (−/−) cells. In contrast, response to diabetic HDL was impaired in both types suggesting diminished effectiveness of diabetic HDL on EC proliferation and migration might be due to the down regulation of SR-BI. Additionally, SR-BI down regulation diminishes diabetic HDL’s capacity to activate Akt chronically.

**Conclusions/Significance:**

Diabetic HDL was dysfunctional in promoting EC proliferation, migration, and adhesion to matrix which was associated with the down-regulation of SR-BI. Additionally, SR-BI down regulation diminishes diabetic HDL’s capacity to activate Akt chronically.

## Introduction

Lipid metabolism disorders associated with type 2 diabetes, such as increased levels of low-density lipoprotein (LDL) and reduced levels of high-density lipoprotein (HDL), have been linked to multiple biological processes implicated in the development of atherosclerosis [1]. The risk of atherosclerosis and coronary heart disease is inversely related to circulating levels of HDL cholesterol and the major HDL protein apolipoprotein A-I (apoA-I) [2]. HDL classically functions in reverse cholesterol transport (RCT), removing cholesterol from peripheral tissues including vessels and delivering it to the liver for final excretion in bile [3]. Along with this, HDL has multiple endothelial actions that also afford cardiovascular protection, including antioxidant, anti-inflammatory, anti-apoptotic, and anti-thrombotic activities [4]. Further, HDL activates endothelial nitric oxide synthase [5], enhances endothelial progenitor cell (EPC) mediated endothelium repair, and stimulates EC proliferation and migration [6]. Additionally, increasing the levels of HDL by drugs, gene therapy, or direct infusion has been shown to improve outcomes in diabetic animal models or in diabetic patients [7], [8].

We previously described that HDL can become dysfunctional in RCT due to oxidative modifications which is increased in subjects with cardiovascular disease (CVD) [9]. HDL from diabetic subjects (D-HDL) has diminished anti-oxidative activity [10] and reduced capacity to stimulate EC NO production and promote EPC-mediated endothelial early repair [11], [12]. Decreased plasma HDL cholesterol concentration in diabetic patients is associated with endothelial dysfunction, which may play a central role in the development of cardiovascular and cerebrovascular diseases [13]. HDL’s effect on endothelial function might be mediated by scavenger receptor class B type I (SR-BI) located on cell membrane and activation of signaling pathways [5], [14]. However, the effect of this pathway in human diabetes has not been investigated systematically.

In the present study, we investigated whether D-HDL has altered function. We found that D-HDL has decreased capacity to stimulate human umbilical vein endothelial cells (HUVECs) proliferation, migration, and adhesion to extracellular matrix (ECM) compared to HDL from normal subjects (N-HDL). These EC responses to HDL are critical for endothelial repair and maintenance of vascular integrity. We demonstrated that these effects of D-HDL are associated with decreased expression of endothelial SR-BI. We utilized mouse aortic endothelial cells (MAECs) derived from wild type (SR-BI +/+) or SR-BI deficient (SR-BI −/−) mice, and tested the responses with N-HDL and D-HDL. The alteration of the signal pathways induced by D-HDL compared to N-HDL suggests a critical role for SR-B1 in mediating the deleterious effects of D-HDL.

## Methods

### Ethics Statement

Healthy control subjects and patients with type 2 diabetes were recruited. Each participant gave written, informed consent after the nature of the procedure was explained. Human umbilical vein endothelial cells (HUVECs) were isolated by collagenase digestion of umbilical veins [15] from fresh cords which were from donors with written informed consent. The study protocol was approved by the Institutional Review Board of The Military General Hospital of Beijing. All animal experimental procedures were approved by the Ethics Committee of Animal Research, Peking University Health Science Center, and the investigation conformed to the Guide for the Care and Use of Laboratory Animals published by the US National Institutes of Health (NIH Publication No. 85-23, revised 1996).

### Materials

Antibodies for western blotting against SR-B1 and β-actin were from Abcam, USA. Antibody against Phospho-Akt (Ser473) was from Cell Signaling Technology, USA. Antibody against Akt1/2 was from Goldbrige, China. Antibody against apolipoprotein A-1 was from Diasorin, USA. HRP-donkey-anti-goat IgG and HRP-goat-anti-rabbit IgG were from Proteintech, China. BrdU proliferation ELISA kit was from Roche Applied Science, Germany. Annexin V-FITC Apoptosis Detection Kit was from Biosea Biotechnology Co., China.

### Subjects

Each patient volunteer underwent a medical history, physical examination, screening laboratory tests and a 75 g oral glucose tolerance test. Patients were stable on antihypertensive, hypoglycemic, and lipid-lowering medications. There was no treatment change during the study. The healthy subjects had no family history of diabetes and had normal glucose tolerance. Clinical and laboratory characteristics of the study participants are shown in the table 1.

**Table 1:** Clinical and laboratory characteristics.

	Healthy Subjects	Diabetic Patients	P value
	(n = 52)	(n = 75)	
Age, y	47±2.0	64±2.0	<0.01
Sex, male/female	34/18	40/35	>0.07
Fasting glucose, mmol/L	5.2±0.1	8.4±0.4	<0.01
TC, mmol/L	5.0±0.1	4.5±0.2	0.05
HDLc, mmol/L	1.4±0.05	1.2±0.05	0.02
LDLc, mmol/L	2.9±0.1	2.8±0.1	0.73
TG, mmol/L	1.2±0.1	1.7±0.1	0.01

Data are presented as mean±SEM as indicated.

TC = total cholesterol; HDLc = high density lipoprotein cholesterol; LDLc = low density lipoprotein cholesterol; TG = triglyceride.

### Lipoprotein Preparation

Blood was drawn in EDTA tubes from healthy volunteer donors and patients with type 2 diabetes after a 12-hour fast. About 2 ml plasma was separated by centrifugation at 2500 rpm at room temperature for 15 minutes. LDL (1.019–1.063), and HDL (1.063–1.210) were isolated from fresh plasma from each participant by ultracentrifugation as previously described [16], and dialyzed with 0.01 M PBS (0.1% EDTA-Na_2_, 100 µM diethylenetriamine pentaacetic acid (DTPA) (Sigma, USA) at 4°C in the dark for 48 h. After that, lipoproteins from each individual were sterilized through a 0.22 µm filter and stored individually in sealed tubes at 4°C in the dark. The purity of HDL was evaluated by SDS-PAGE and western blotting using anti-apoA-I polyclonal antibody (DiaSorin, USA), and the level of apoA-I in HDL was quantified by nephelometry (Dimension XPand, Dade Behring, Germany). The lipoproteins were used within 1 month after separation. To create glycated HDL, fresh human HDL (5 mg protein) was incubated with 25 mmol/L glucose in PBS under sterile conditions for 1 week at 37°C (referred to as G-HDL) [17]. Oxidative modification of HDL was performed by dialysis at lipoprotein concentration of 0.8 mg protein/ml against PBS containing 5 µM CuSO4 for 24 hours at 37°C (referred to as OX-HDL) [18], and dialysis against PBS for 48 hours at 4°C to remove the copper ion.

### Animals

SR-BI +/+ or SR-BI −/− mice were obtained from Dr. George Liu (Peking University). Experiments were performed with sex-matched 3-month-old SR-BI +/+ and SR-BI −/− mice. Genotyping was done using genomic DNA extracted from tails by PCR. Both mutants and control animals were littermates from heterozygous crosses. In all experiments, both male and female mice were used in approximately equal ratios.

### Cell Cultures and Treatments

HUVECs were isolated as described previously [15] and cultured in Endothelial Cell Medium (Sciencell, USA) consisting of basal medium, 5%(V/V) fetal bovine serum, 1%(V/V) endothelial cell growth supplement and 1%(V/V) penicillin/streptomycin solution, in a humidified atmosphere (5% CO_2_) at 37°C. HUVECs were pooled and used at passages 1–5. To isolate MAECs, mouse aortas were excised under anaesthesia with sodium pentobarbital (50 mg/kg, i.p.). The adequacy of anaesthesia was monitored through pinching the hind paw, and the sufficiently sedated mice were euthanized through cervical dislocation. Two mm sections of mouse aortas were placed on matrigel pre-coated plates and cultured in Endothelial Cell Medium at 37°C for 7–14 days to promote MAEC outgrowth prior to passaging. MAECs were used at passage 2 to 3 and were stained with anti-CD31 and CD34 antibodies to confirm endothelial cells phenotype (Data not shown).

### Cell Proliferation by Tetrazolium-based Colorimetric Assay (MTT assay)

HUVECs or MAECs were plated with Endothelial Cell Medium and 1% fetal bovine serum at a density of 4000 cells/well in 96-well plates and cultured overnight. And then, cells were treated with N-HDL or D-HDL for 24 hours at the indicated apoA-I concentration. Four hours before the end of the incubation, 20 µl of 5 mg/ml 3-(4,5-Dimethylthiazol-2-yl)-2,5-diphenyltetrazolium bromide (MTT) was added to each well [15]. Then, 100 µl/well DMSO was added and the optical density of the soluble formazan was read at 570 nm with an ELISA plate reader (model 550, BioRad, USA).

### BrdU Proliferation Assay

HUVECs were plated with Endothelial Cell Medium and 1% fetal bovine serum at a density of 1000 cells/well in 96-well plates and cultured overnight. And then, HUVECs were treated with N-HDL, D-HDL, G-HDL or Ox-HDL for 12, 24, 36, 48, 60, 72 hours at 100 µg/ml apoA-I concentration. The cells were labeled with 20 µl/well of BrdU labeling solution as described previously [19], and then incubated with 200 µl/well of FixDenat. After incubated with 100 µl/well of Anti-BrdU-POD working solution for 90 minutes and washed 3 times, substrate solution (TMB) was added and the absorbance of each well was read at 450 nm with an ELISA plate reader (model 550, BioRad, USA).

### Cell Apoptosis Assay

Cell apoptosis assay was performed as described previously [20]. HUVECs were seeded at a density of 5×10^5^ cells/well in 6-well plates and cultured overnight. And then, HUVECs were treated with N-HDL, D-HDL, G-HDL or Ox-HDL at 100 µg/ml apoA-I concentration for 24 hours. Cells were trypsinized, washed with cold PBS for 3 times, resuspended with 200 µl binding buffer, and then incubated with 10 µl Annexin V-FITC for 15 min at room temperature in the dark. After that, 300 µl binding buffer and 5 µl propidium iodide were added and cell apoptosis was quantified by flow cytometry in a FACS Calibur (BD Bioscience, Bedford, USA).

### Endothelial Cell Migration Assays

#### Wound healing

HUVECs were plated with Endothelial Cell Medium and 5% bovine serum in 24-well plate (5×10^5^/ml cells/well). And then, HUVECs were serum deprived and scratched, treated with HDL (normal or diabetic) at an apoA-I concentration of 100 µg/ml [21]. 24 hours later HUVECs were fixed with methanol, stained with hematoxylin-eosin stain, and viewed under an inverted microscope (Nikon, Japan). The cells which had migrated past the wound edge were photographed and quantified in 10 random high power (100×) fields. Results were confirmed in at least three independent experiments.

#### Transwell experiments

Quantitative migration assays with HUVECs or MAECs were performed using a modified Boyden chamber (Minicell, Millipore, USA) as described previously [21]. The lower face of the polycarbonate filter (Transwell insert) was coated with 10 µg of fibronectin for 30 minutes. The Transwell insert was placed back to the 24-well plate and the lower chamber was filled with 0.6 ml of Endothelial Cell Medium and 5% bovine serum. HUVECs or MAECs (5000 cells/well) in Endothelial Cell Medium were plated into the upper chamber. N-HDL or D-HDL was individually added to the upper chamber at apoA-I concentration of 100 µg/ml. After 8 hours incubation, all non-migrated cells were removed from the upper face of the Transwell membrane with a cotton swab, and migrated cells were fixed and stained with hematoxylin-eosin stain. Cell migration was quantified by counting the number of stained cells per in 10 random high power (100×) fields photographed for each chamber.

### EC-ECM Adhesion Assay

HUVECs pretreated with HDL were incubated in wells coated with Matrigel Membrane Matrix [22]. Wells of 96-well plates were coated with 20 µl of 0.1 mg/ml Matrigel Membrane Matrix (Vigorous Biotechnology Beijing Co., China) in PBS at room temperature and allowed to air dry. The HUVECs were serum deprived and pretreated with N-HDL or D-HDL at a final apoA-I concentration of 100 µg/ml for 24 hours before plating into each well. The plates were blocked with 20 µl 2% BSA at 37°C for 1 hour, and then washed with PBS. The pretreated HUVECs in Endothelial Cell Medium with 0.1% BSA were plated in each well at a density of 8×10^4^ cells/well for 1 hour at 37°C. Thereafter, nonadherent cells were removed by washing with PBS 3 times and 40 µg/well of MTT was added for 4 hours at 37°C. After discarding MTT and adding 200 µl DMSO, the relative number of adherent cells was quantified by measuring the absorption at 570 nm with an ELISA plate reader (model 550, BioRad, USA).

### RNA Isolation and Real-time Quantitative PCR Experiments

Total RNA was isolated from HUVECs using Trizol Reagent kit (Invitrogen, USA) and then was reversely transcribed to Rnase-treated cDNA using a First-Strand cDNA synthesis kit (TransGen Biotech, China). Real-time PCR was taken using SYBR Green PCR Master Mix (Applied Biosystems, UK) in a real-time PCR system – Opticon (MJ Research, USA). The sequence of the PCR forward primer for SR-BI: 5′ CTTCCTCGAGTACCGCAC 3′, reverse primer for SR-BI: 5′ CTTCCTCGAGTACCGCAC 3′. And the sequence of the PCR forward primer for Glyceraldehyde-3-phosphate dehydrogenase (GAPDH): TGAAGGTCGGAGTCAACGGATTTGGTCGTA, reverse primer for GAPDH: ATCTCGCTCCTGGAAGATGGTGATGGGATT. The level of SR-BI mRNA was normalized to the relative ratio of GAPDH mRNA. Each RT-PCR assay was performed 3 times, and the results are shown as the mean±SEM.

### Integrins Flow Cytometry Assay

HUVECs were treated with normal N-HDL or D-HDL at apoA-I concentration of 100 µg/ml for 6 hours, and then cells were labeled with a PE mouse anti-human integrin β1 and FITC mouse anti-human integrin αv IgG, or isotype IgG respectively (eBioscience, USA) and were analyzed on FACS Calibur flow cytometer (Becton Dickinson, NJ, USA) which analyses 10,000 cells for the presentation of specific antigen.

### Western Blotting

Proteins from cell lysates were separated by SDS-PAGE [21]. The expression of endothelial SR-BI, ABCG1, Akt-phosphorylation, and Akt1/2 were analyzed by Western blot. HUVECs were serum deprived and treated with media or different types of HDL at an apoA-I concentration of 100 µg/ml for the indicated times. After the treatment, cells were lysed in a buffer containing 50 mM Tris (pH 7.4), 150 mM NaCl, 1% NP-40, 0.5% sodium deoxycholate, 0.1% SDS, 0.1% EDTA, 1% Triton X100, and 1× protease inhibitors (Roche, Switzerland). Cell debris was removed by centrifugation (12000 g for 5 minutes), and the protein concentration was determined by Coomassie brilliant blue method. Cell lysates were analyzed by western blot using specific antibodies described above. Cell lysates (120 to 150 µg protein per lane) were subjected to electrophoresis on 10% SDS-polyacrylamide gels (SDS-PAGE) and transferred onto nitrocellulose membranes (Pall Corporation, USA) according to standard procedures. The membranes were blocked for 2 hours with 5% non-fat milk containing 0.05% Tween 20 in Tris buffered saline, and then were incubated with each primary antibody (1∶500–1∶2000 dilution) overnight at 4°C followed by the appropriate HRP-conjugated secondary antibody diluted according to the manufactures’ protocol. Antibody binding was detected using the SuperSignal West Pico Kit (Pierce, USA) according to the manufacturer’s instructions and quantified using Adobe Photoshop software.

### Determination of Cell Surface Expression of SR-BI by Enzyme Immunoassay

HUVECs in 96-well plates were grown till 70% confluency. HUVECs were serum deprived and treated with N-HDL or D-HDL in serum-free medium for 6 hours at 37°C. Cells were washed with PBS, fixed with 1% paraformaldehyde for 15 minutes, and then washed with PBS 3 times and blocked with 2% BSA for 2 hours at 37°C. After that, cells were incubated with monoclonal antibody for SR-BI (1∶400; Abcam, Hong Kong) and followed by secondary binding with a HRP-conjugated antibody (1∶1000; Boster, China) as described previously [23]. SR-BI expression on cell surface was quantified by measuring the colorimetric conversion of 3,3,5,5-tetramethylbenzidine at 450 nm using TMB peroxidase EIA substrate kit (Bio-Med innovation, China).

### Statistical Analysis

All experiments were performed in triplicate if not mentioned. The results of multiple observations are presented as the mean ± SEM or as a representative result of more than three different separate experiments, unless otherwise stated. Data were analyzed with GraphPad Prism (GraphPad Prism Software, USA), using the statistical test stated in the figure legends; and, values were considered significant at *p*<0.05.

## Results

### Diabetic HDL has Reduced Capacity to Stimulate EC Proliferation

**Figure 1:**
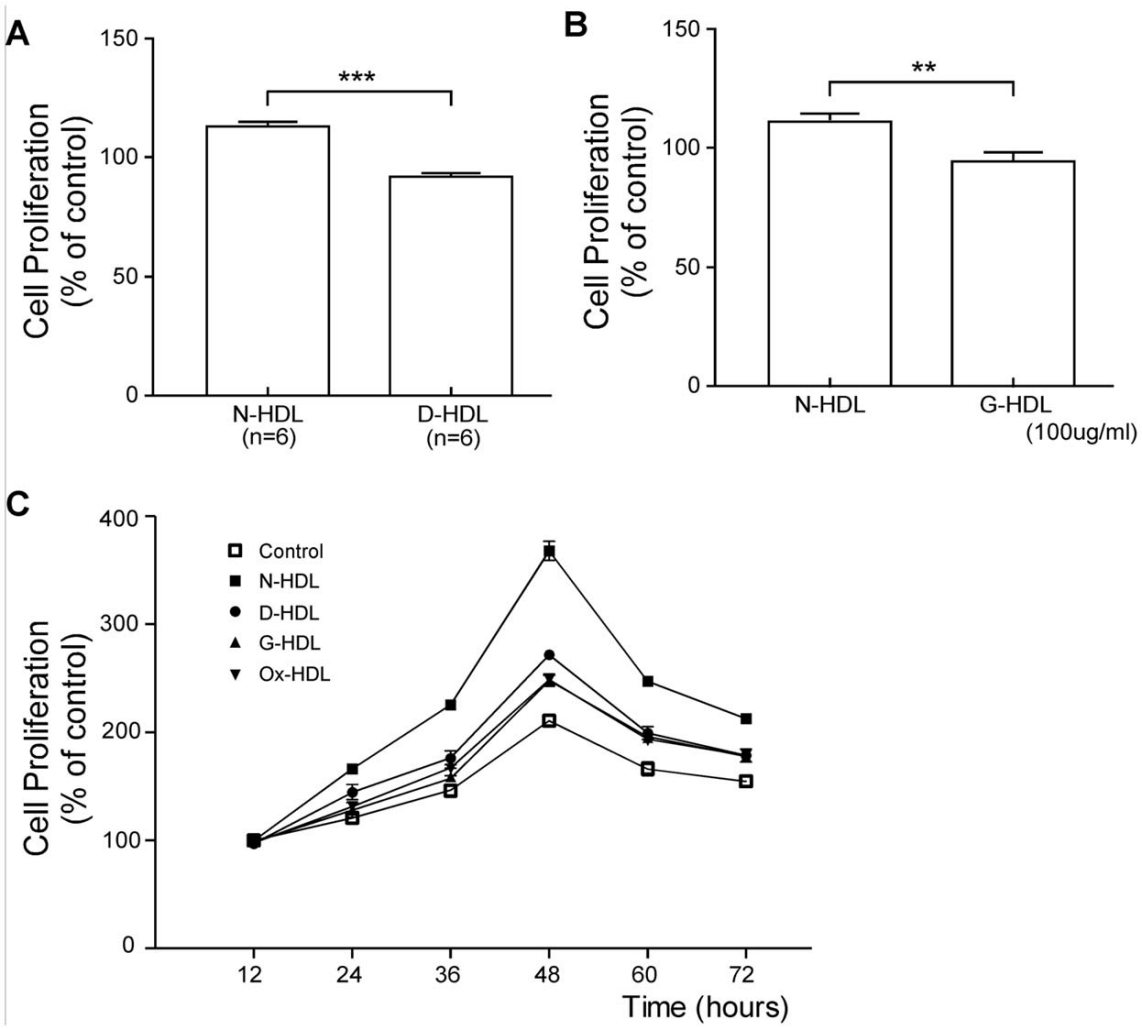
Diabetic HDL is less efficient in stimulating EC proliferation. A) HUVECs were treated with N-HDL and D-HDL for 24 hours, and cell proliferation was measured using MTT assay. Mean ± SEM, n = 6, ***, *p*<0.001 by student’s t-test. **B)** HUVECs were treated with N-HDL or G-HDL for 24 hours at apoA-I concentrations of 50 and 100 µg/ml respectively, and cell proliferation was measured using MTT assay. Although N-HDL promoted cell proliferation at 24 hours, G-HDL at 100 g/ml apoA-I decreased cell proliferation (mean ± SEM; **, *p*<0.01 by student’s t-test). C) HUVECs were treated with N-HDL, D-HDL, G-HDL or Ox-HDL for 12, 24, 36, 48, 60 or 72 hours at apoA-I concentrations of 100 µg/ml and cell proliferation using BrdU proliferation assay was shown (mean ± SEM; **, p<0.01 by ANOVA and Bonferroni’s Multiple Comparison Test).

HUVECs were used to test HDL-induced cell proliferation. While N-HDL led to increased cell number, D-HDL has 18% less effect compared to N-HDL (Fig. 1A, n = 6, p<0.001 comparing N-HDL vs. D-HDL). The incubation of N-HDL (100 µg/ml apoA-I) led to a small increase in relative cell number. In contrast, we observed a decrease in cell number with this concentration of G-HDL (Fig. 1B, *p*<0.01 comparing N-HDL vs. G-HDL). A cell growth curve of HUVECs examined using BrdU assay showed that the cell number increased during 12 to 48 hours, but decreased during 48 to 72 hours. Although N-HDL, D-HDL, G-HDL and Ox-HDL can promote the proliferation of EC during 12 to 72 hours culture, all D-HDL, G-HDL and Ox-HDL stimulated HUVEC proliferation much less efficiently compared to N-HDL (Fig. 1C). Cell apoptosis assay showed that there was no significant difference among N-HDL, D-HDL, G-HDL, Ox-HDL treated cells and control cells (Figure S1).

### Diabetic HDL has Reduced Capacity to Stimulate EC Migration

**Figure 2:**
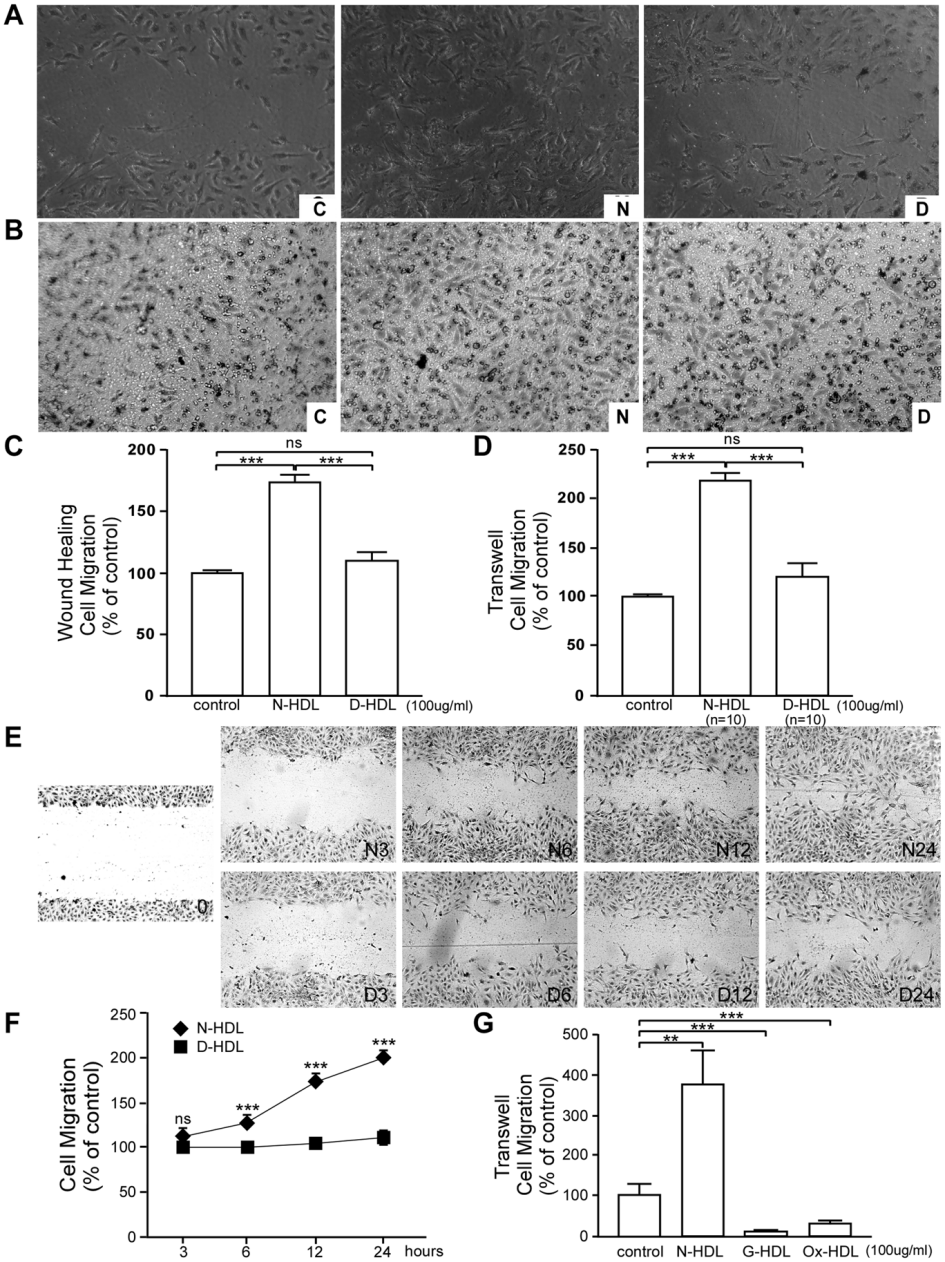
Diabetic HDL is much less efficient in promoting EC migration. A) Scratched HUVEC monolayers were treated with media alone (C, control), N-HDL (N), or D-HDL (D) for 24 hours, and migration into the wound was photographed (100× objective lens). **B)** N-HDL or D-HDL (n = 10 each) was added to each well and transwell migration was evaluated after 8 hours. **C)** Quantification migration in the wound healing assay (n = 3, mean ± SEM; ***, *p*<0.001 by ANOVA and Bonferroni’s Multiple Comparison Test). **D)** Cell migration based upon an 8 hour incubation in the transwell migration assay (n = 10, mean ± SEM, ns, *p*>0.05 and ***, *p*<0.001 by ANOVA and Bonferroni’s Multiple Comparison Test) was shown. **E)** Scratched HUVEC monolayers were treated with N-HDL (N), or D-HDL (D) for 3, 6, 12 and 24 hours, and the migration into the wound was photographed (100× objective lens). **F)** The migration in wound healing assay was quantified (n = 3, mean ± SEM; ***, *p*<0.001 by ANOVA and Bonferroni’s Multiple Comparison Test). **G)** Transwell migration assay was applied to HUVECs treated with media alone (C, control), N-HDL, G-HDL, or Ox-HDL at an apoA-I concentration of 100 µg/ml for 8 hours (mean ± SEM, **, *p*<0.01 and ***, *p*<0.001 by ANOVA test and Bonferroni’s Multiple Comparison Test).

N-HDL increased HUVEC migration into the wound area by ∼70% vs. the untreated control; however, D-HDL did not increase migration above the untreated control level (Fig. 2A, C; p<0.001 comparing N-HDL vs. control and D-HDL, *p>*0.05 for D-HDL vs. control). We incubated HUVECs with individual HDL (n = 10 each). N-HDL increased median HUVEC migration to ∼220% of the control value, while D-HDL only increased to ∼120% of the control value (Fig. 2B, D; *p*<0.001 for N-HDL vs. control and D-HDL, *p>*0.05 for D-HDL vs. control). Furthermore, HUVEC were scratched and treated with N-HDL or D-HDL for 0, 3, 6, 12, 24 hours (Fig. 2E, F). Both HDLs stimulated HUVEC migration in a time-dependent manner. However, compared to N-HDL, D-HDL did not stimulate HUVEC migration. We previously found that glycation and oxidation levels are much higher in diabetic HDL [21]. To determine the effect of G-HDL or Ox-HDL on EC migration, HUVECs were incubated with control media, G-HDL, or Ox-HDL for 8 hours. Transwell migration assay showed that both G-HDL and Ox-HDL can inhibit cell migration (Fig. 2G). Taken together, N-HDL promoted the migration of EC, while D-HDL did not.

### Diabetic HDL has Reduced Capacity to Promote EC Adhesion to ECM

**Figure 3:**
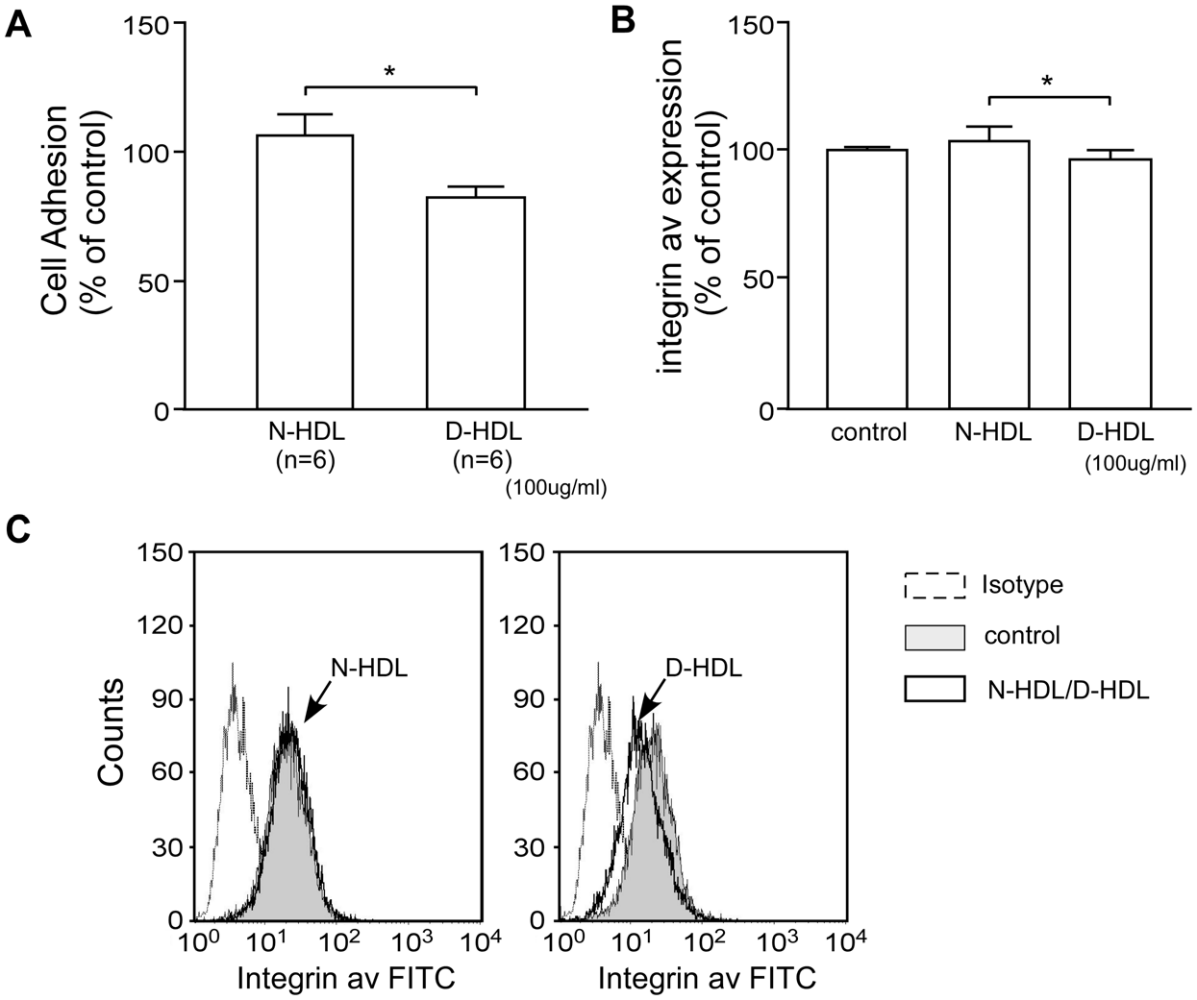
Diabetic HDL decreases cell adhesion to ECM. A) HUVECs were pretreated with N-HDL or D-HDL for 24 hours, and relative cell adhesion to ECM was determined after a 1 hour incubation, n = 6, mean ± SEM, *, *p*<0.05 by student’s t-test. **B)** HUVECs were pretreated with N-HDL or D-HDL for 6 hours. Integrin αv expression was determined by flow cytometry, n = 3, mean ± SEM, *, p<0.05 by student’s t-test. **C)** Flow cytometry overlay histogram shows integrin αv expression of HUVECs treated with N-HDL or D-HDL compared to control.

We observed significantly decreased adhesion of HUVECs to ECM after D-HDL pretreatment compared to N-HDL (Fig. 3A, n = 6 each). We examined the expression of integrin β1 and integrin αv of HUVECs by flow cytometry after 6 hour treatment with N-HDL or D-HDL. Integrin β1 did not show significant difference, while integrin αv was down-regulated by D-HDL (Fig. 3B, C; *p*<0.05 for N-HDL vs. D-HDL).

### Diabetic HDL Down-regulates the Expression of SR-BI in EC and on Cell Surface

**Figure 4:**
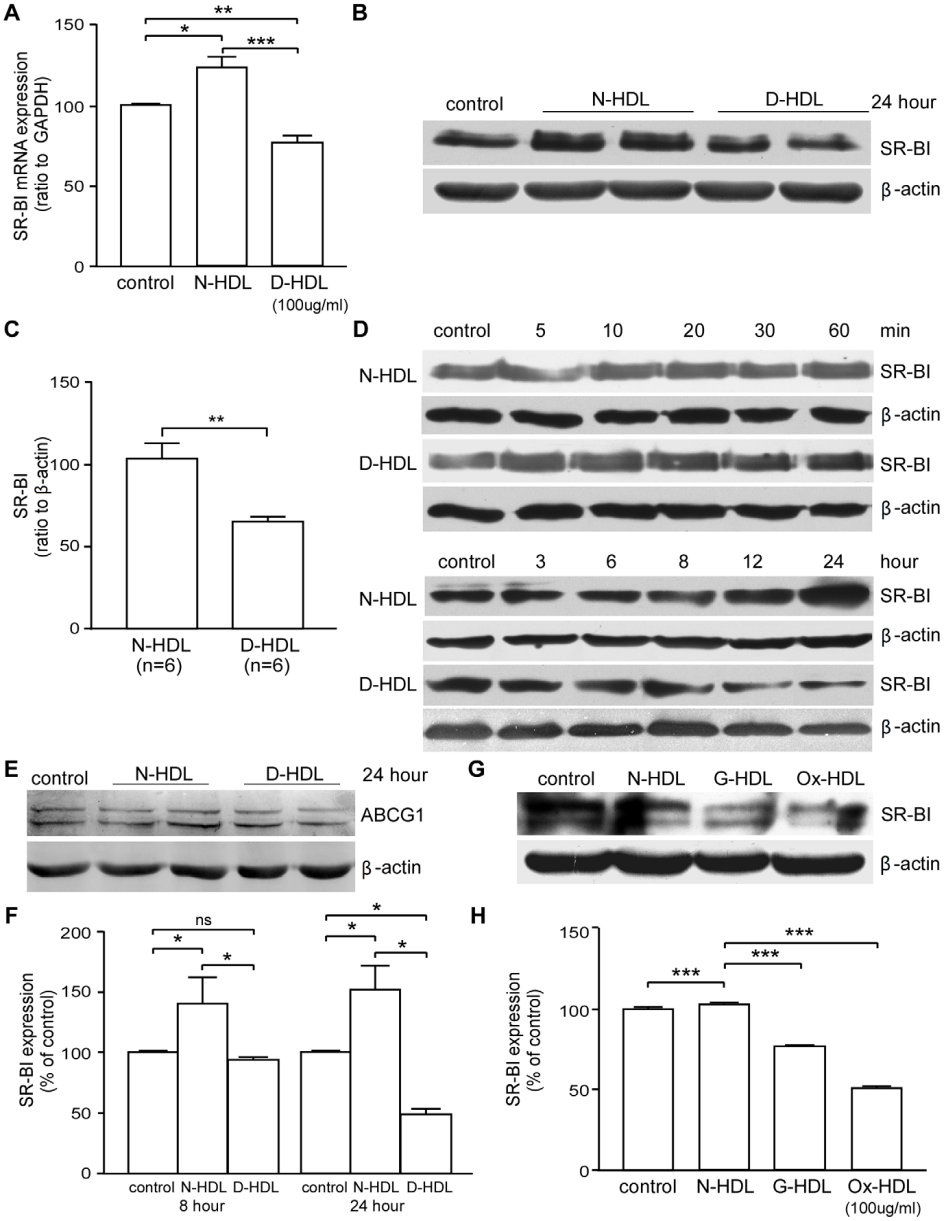
EC SR-BI expression is reduced by diabetic HDL. A) HUVECs were treated with PBS, N-HDL or D-HDL for 12 hours. Real-time PCR experiment shows that the gene expression of SR-BI, and the ratio of SR-BI/GAPDH (mean ± SEM) is shown, n = 6 each, ***, *p*<0.001 by one-way ANOVA. **B)** HUVECs were treated with PBS, N-HDL or D-HDL for 24 hours. Representative western blot shows that D-HDL down-regulated the expression of SR-BI. **C)** The density of the SR-BI bands was normalized to the density of the β-actin band. The ratio of SR-BI/β-actin (mean ± SEM) is shown, *p*<0.01 by student’s t-test. **D)** HUVECs were treated with N-HDL or D-HDL for 5, 10, 20, 30, 60 minutes, 3, 6, 8, 12, or 24 hours, and SR-BI expression was shown. **E)** HUVECs were treated with N-HDL or D-HDL for 24 hours, and ABCG1 expression was shown. **F)** HUVECs were treated with N-HDL or D-HDL for 8 or 24 hours. SR-BI levels on the cell surface were shown as percentage of control, mean ± SEM, *, P<0.05 by one-way ANOVA. **G)** HUVECs were treated with with media alone (C, control), N-HDL, G-HDL, and Ox-HDL at an apoA-I concentration of 100 µg/ml for 24 hours, and western blotting assay was performed. **H)** The density of the SR-BI bands was normalized to the β-actin band (***, *p*<0.001 by a Student’s t test).

SR-BI has been reported to play a key role in mediating the effect of HDL on promoting EC migration and monolayer integrity *in vitro* and *in vivo* [5], [14]. D-HDL treatment led to ∼44% decreased SR-BI mRNA expression of HUVECs compared to N-HDL treatment (Fig. 4A; *p*<0.001). And it showed lower protein expression of SR-BI after treated with D-HDL (Fig. 4B). As shown, D-HDL treatment led to ∼32% decreased SR-BI expression compared to N-HDL treatment (Fig. 4C; n = 6, *p*<0.01). Further, we examined the levels of cellular SR-BI after 5, 10, 20, 30, 60 minutes, 3, 6, 8, 12, or 24 hours treatment with N-HDL or D-HDL. Both N-HDL and D-HDL didn’t show differential regulation of SR-BI expression for 5, 10, 20, 30, or 60 minutes treatment. Over longer times of exposure, N-HDL up-regulated SR-BI expression while D-HDL had the opposite effect (Fig. 4D). To determine whether ABCG1, an important cholesterol transporter [24], was involved in functional deficiency of D-HDL, we examined the levels of cellular ABCG1 by Western blot. There was no significant difference between N-HDL and D-HDL treatments (Fig. 4E). The abundance of SR-BI on plasma membrane was investigated using cell ELISA. N-HDL treatment for 8 hours induced elevated expression of SR-BI on HUVEC surface to ∼139% of the control value, but D-HDL failed to do so (Fig. 4F; *p*<0.05 for N-HDL vs. control and D-HDL). Further, 24-hour treatment with D-HDL resulted in significant down-regulation in SR-BI expression on HUVEC surface compared with N-HDL or no treatment (Control) (Fig. 4F). Meanwhile, both G-HDL and Ox-HDL down regulated the SR-BI expression of HUVEC (Fig. 4G, H).

### HDL’s Effects on EC Proliferation and Migration are Dependent on SR-BI

**Figure 5:**
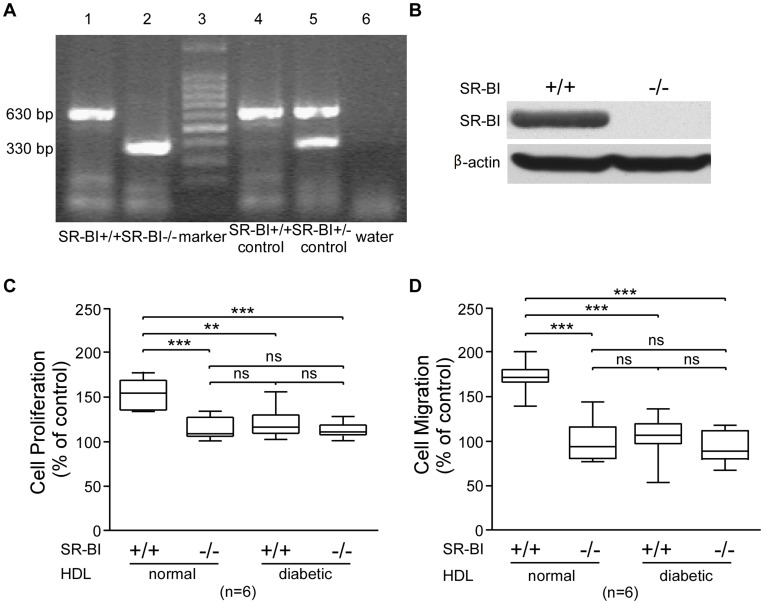
SR-BI mediates the effects of N-HDL on MAEC proliferation and migration. A) Representative agarose gel of SR-BI genotype assay by PCR. Lanes 1 and 2 are derived from 2 littermates from the offspring of SR-BI +/− parents. Lane 3 indicates molecular weight markers. Lanes 4 and 5 are derived from a wild type (SR-BI +/+) and a heterozygous (SR-BI +/−) individual. Lane 6 is negative control with water. **B)** Protein expression of SR-BI in liver from SR-BI (+/+) and SR-BI (−/−) mice was measured using western blot. **C)** MAECs from SR-BI (+/+) and SR-BI (−/−) mice were treated with N-HDL or D-HDL for 24 hours. Relative cell number was compared to untreated control cells using the MTT assay. Box-whisker plots of cell number are shown (**, *p*<0.01 by non parametric ANOVA test, n = 6). **D)** MAECs from SR-BI (+/+) or SR-BI (−/−) mice were treated with N-HDL or D-HDL for 8 hours and transwell migration assays were performed (***, *p*<0.001 by non parametric ANOVA test, n = 6).

Representative agarose gel of SR-BI genotype assay by PCR was shown (Fig. 5A). And lack of SR-BI expression in the SR-BI (−/−) MAEC was confirmed by western blot (Fig. 5B). The addition of N-HDL to SR-BI (+/+) MAECs led to a 1.54-fold increase in cell number vs. the control; however, N-HDL addition to SR-BI (−/−) MAECs only led to a 1.15-fold increase in cell number (Fig. 5C, *p*<0.01, Bonferroni’s Multiple Comparison Test). Thus, the majority of the effect of N-HDL on EC proliferation was SR-BI dependent. In contrast, the addition of D-HDL to SR-BI (+/+) or SR-BI (−/−) MAECs led to a similar and modest increase in cell number vs. control, not significantly different from the increase observed by N-HDL addition to SR-BI (−/−) MAECs (Fig. 5C, *p*>0.05, Bonferroni’s Multiple Comparison Test). Similar results were obtained using transwell cell migration assay. N-HDL addition led to a 1.7-fold increase in cell migration in SR-BI (+/+) MAEC vs. control, but no change in SR-BI (−/−) MAECs vs. control. D-HDL addition to either SR-BI (+/+) or SR-BI (−/−) MAECs did not lead to any induction of cell migration vs. control, and were significantly different to cell migration induced by N-HDL addition to SR-BI (+/+) cells (Fig. 5D, p<0.001, Bonferroni’s Multiple Comparison Test). Thus, the loss of function of D-HDL in mediating cell proliferation and migration is consistent with the D-HDL induced loss of cellular SR-BI protein.

### Diabetic HDL has Diminished Capacity to Activate Akt Chronically

**Figure 6:**
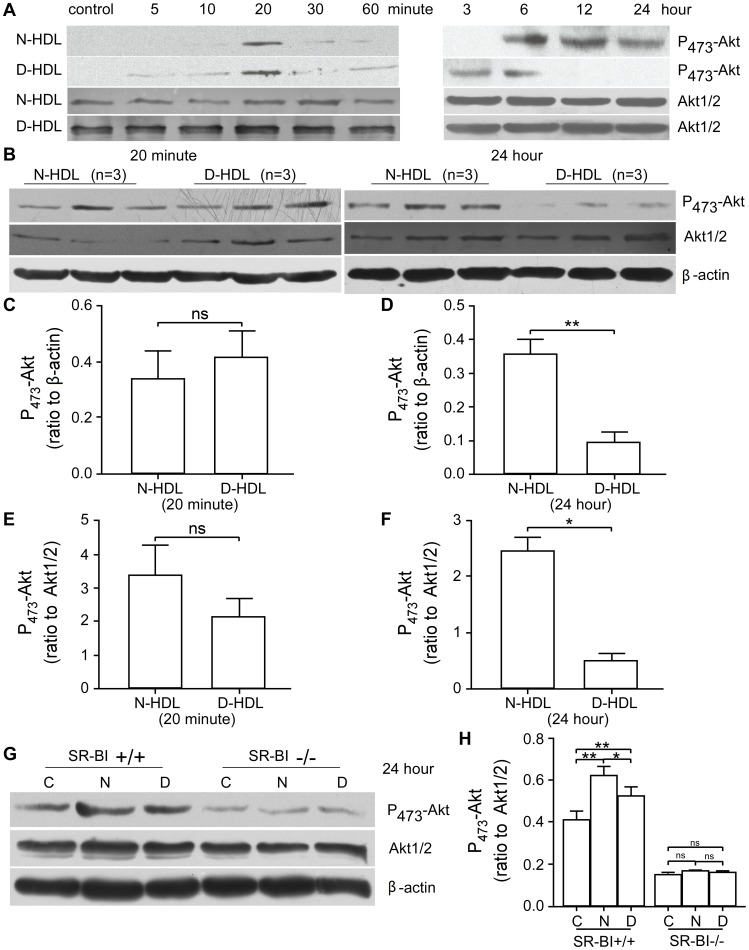
Akt phosphorylation induced by HDL. A) HUVECs were treated without (control) or with the addition of N-HDL or D-HDL for 5, 10, 20, 30, 60 minutes, 3, 6, 12, or 24 hours. Expression levels of phospho-Akt (Ser473) and Akt1/2 were analyzed by western blotting. **B)** HUVECs were treated with N-HDL or D-HDL (n = 3 each) for 20 minutes or 24 hours. Expression levels of phospho-Akt (Ser473), Akt1/2, and β-actin were analyzed by western blotting. **C)** The density of the phospho-Akt bands at 20 minutes was normalized to the β-actin band (ns, *p*>0.05). **D)** The density of the phospho-Akt bands at 24 hours was normalized to the β-actin band (**, *p*<0.01 by a student’s t test). **E)** The density of the phospho-Akt bands at 20 minutes was normalized to the Akt1/2 band (ns, p>0.05). **F)** The density of the phospho-Akt bands at 24 hours was normalized to the Akt1/2 band (*, p<0.05 by a student’s t test). **G)** MAECs from SR-BI (+/+) or SR-BI (−/−) mice were treated without (control) or with N-HDL or D-HDL for 24 hours, and expression levels of phospho-Akt (Ser473), Akt1/2, and β-actin were analyzed by western blotting. **H)** The density of the phospho-Akt bands of MAECs from SR-BI (+/+) or SR-BI (−/−) mice was normalized to the Akt1/2 band (*, p<0.05 and **, p<0.01 by a student’s t test).

Akt phosphorylation has been demonstrated to play a role in the signal transduction leading to EC migration [5], [14]. To further describe the mechanisms by which D-HDL is dysfunctional in stimulating EC migration, we determined whether the Akt phosphorylation was affected during this process. HUVECs were treated with N-HDL or D-HDL for 5, 10, 20, 30, 60 minutes, 3, 6, 12, or 24 hours and analyzed using western blotting assay. Both HDL preparations led to Akt phosphorylation with the similar appearance of phosphorylation at 20 minutes (Fig. 6A). The late phase of Akt phosphorylation by N-HDL addition was robust and persisted from the 6 to 24 hour time points; however, the late phase in response to D-HDL was diminished such that there was dramatically reduced phosphorylation at the 12 and 24 hour time points (Fig. 6A). This result was confirmed and quantified in a separate study in which HUVECs were treated with N-HDL or D-HDL individually (n = 3 each) for 20 minutes or 24 hours. While the level of Akt phosphorylation was equivalent after 20 minutes, there was markedly less Akt phosphorylation 24 hours after the addition of D-HDL compared to N-HDL (Fig. 6B, C, D, E, F; p<0.01 by unpaired t-test). To confirm the results, MAECs from SR-BI (+/+) or SR-BI (−/−) mice were treated with N-HDL or D-HDL for 24 hours. There was less Akt phosphorylation after the addition of D-HDL compared to N-HDL in wild type cell. And, there were no prominent inductions of Akt phosphorylation in SR-BI (−/−) cell in response to N-HDL and D-HDL addition (Fig. 6G, H).

## Discussion

Clinical studies demonstrate that increased levels of HDL have beneficial effects on atherosclerosis progression [2]. HDL has multiple endothelial actions which afford cardiovascular protection [4], [6], among which, EC proliferation and migration may play a crucial role in vascular self-repair. Decreased plasma HDL cholesterol concentration in diabetic patients is frequently associated with endothelial dysfunction, and clinical studies demonstrate that increased levels of HDL can reduce the risk of atherosclerosis progression in diabetic patients [7], [8]. Furthermore, D-HDL has impaired ability to activate eNOS, and EPC related early repair [11], [25]. However, direct demonstration that D-HDL is dysfunctional in stimulating EC proliferation and migration has not yet been reported.

EC monolayer integrity is maintained through the replacement of damaged cells via proliferation and migration of neighboring cells [26], or via repopulation with blood-borne EPCs [27]. HDL stimulates EC proliferation in a process that is dependent upon calcium [28]. The present study demonstrates that N-HDL enhances the proliferative activity of EC; however, compared to N-HDL, all D-HDL, G-HDL and Ox-HDL had reduced proliferative capacity. We previously found that glycation and oxidation levels are much higher in D-HDL [21]. ApoA-I glycation has been identified in the plasma of diabetic patients [29],and glycation forms advanced glycation end products (AGEs). Also, *in vitro* and animal studies have shown that non-enzymatic glycation impairs the anti-inflammatory and RCT functions of apoA-I [30], providing a potential mechanism for the dysfunctional nature of D-HDL [31]. Not only D-HDL can be transformed to an oxidized form, but also be defective in anti-oxidative activity [10].

HDL stimulates EC migration to a similar extent as basic fibroblast growth factor (bFGF). HDL-mediated migration is independent of cell proliferation, and is mediated via a distinct signaling mechanism [32]. Recent studies indicate that HDL promotes EC migration in an NO-independent manner through the SR-BI-mediated activation of Rac GTPase, and that PDZK1, the SR-BI adaptor protein, is required for this activation [5], [14]. Furthermore, HDL-mediated migration is not prevented by Pertussis toxin (PTX). The present study demonstrates that D-HDL, compared to N-HDL, does not promote EC migration. G-HDL and Ox-HDL have been found with much higher modification level than D-HDL [21]. That could be the reason why G-HDL and Ox-HDL have worse effects in promoting EC migration (Fig. 2G). D-HDL also had diminished capacity to promote EC adhesion to ECM with the down regulation of integrin αv.

Our investigation demonstrates that levels of SR-BI in EC and on cell surface were decreased after a chronic incubation with D-HDL vs. N-HDL. Importantly, modifications such as glycation or oxidation of HDL *in vitro* can down-regulate SR-BI expression, which might implicate such modifications can contribute to its partial function loss. SR-BI protects against early-onset atherosclerosis using SR-BI/apolipoprotein E double homozygous knockout mice [33]. HDL-mediated stimulation of EC migration starts with the interaction between HDL and SR-BI, and thereby the HDL interaction with SR-BI elicits various signaling cascades such as src-kinase, PI3-kinase, Akt, and p38 MAPK; and, these responses are independent of eNOS [4]. We speculate that the down regulation of SR-BI could be the primary mechanism for D-HDL’s diminished capacity for stimulating EC proliferation and migration. This concept is supported by our finding in MAECs, where N-HDL was competent to promote these EC activities in SR-BI (+/+) cells, but not in SR-BI (−/−) cells, while D-HDL was equally inefficient in promoting EC proliferation and migration in both types. Thus, it appears that SR-BI plays a key role in differentiating the effects between N-HDL and D-HDL.

Akt kinase activation is considered as an important mediator in the signal transduction pathway of EC proliferation and migration [14], [34]. The present study demonstrates that both N-HDL and D-HDL led to early Akt phosphorylation at the 20 minute time point, but that only N-HDL led to robust Akt phosphorylation 12 to 24 hours later more than D-HDL. N-HDL led to higher late phase Akt phosphorylation than D-HDL in SR-BI (+/+), but there was no difference of Akt phosphorylation between N-HDL and D-HDL treatment in SR-BI (−/−) MAECs. We speculate that the diminished late phase Akt activation by D-HDL may be due to D-HDL induced down regulation of SR-BI.

The present observations reveal a novel series of mechanisms by which D-HDL is a negative modulator of EC re-endothelialization activities. Our findings provide a new framework for understanding how D-HDL is dysfunctional in promoting vascular protection. These findings also provide a rationale for more research to uncover the specific HDL modifications in diabetic patients and point to the prevention of these HDL modifications as a potential therapeutic target to prevent cardiovascular disease. Also, these modifications including glycation and oxidation of HDL will be used as diagnosing markers for evaluating HDL functions in type 2 diabetes.

## Supporting Information

Figure S1 **HUVEC apoptosis with normal, diabetic, glycated and oxidized HDL treatment. A**. Flow cytometry histograms represent Annexin V-FITC staining in *x* axis and PI in *y* axis. The numbers represent the percentage of early (Annexin V+/PI−) [lower right quadrant] and late (Annexin V+/PI+) [upper right quadrant] apoptotic cells in HUVECs treated with PBS, N-HDL, D-HDL, G-HDL or Ox-HDL for 24 hours. **B.** There was no significant difference in the number of apoptotic cells between control cells and treated cells. N = 3, mean ± SEM, p>0.05 by ANOVA and Bonferroni’s Multiple Comparison Test.
(DOC)
