# Supplementary material for: Diabetic HDL Is Dysfunctional in Stimulating Endothelial Cell Migration and Proliferation Due to Down Regulation of SR-BI Expression
Source: PLoS One. 2012 Nov 2;7(11):e48530. doi: 10.1371/journal.pone.0048530 (PMC3487724; doi:10.1371/journal.pone.0048530)

**Data Supplement Figure Legends**

**Figure 1. HUVEC apoptosis with normal, diabetic, glycated and oxidized HDL treatment.**

**A**. Flow cytometry histograms represent Annexin V-FITC staining in *x* axis and PI in *y* axis. The numbers represent the percentage of early (Annexin V+/PI-) [lower right quadrant] and late (Annexin V+/PI+) [upper right quadrant apoptotic cells in HUVECs treated with PBS, N-HDL, D-HDL, G-HDL or Ox-HDL for 24 hours. **B.**  There was no significant difference in the number of apoptotic cells between control cells and treated cells. N=3, mean ± SEM, p>0.05 by ANOVA and Bonferroni's Multiple Comparison Test.

**Data Supplement Figure 1.**


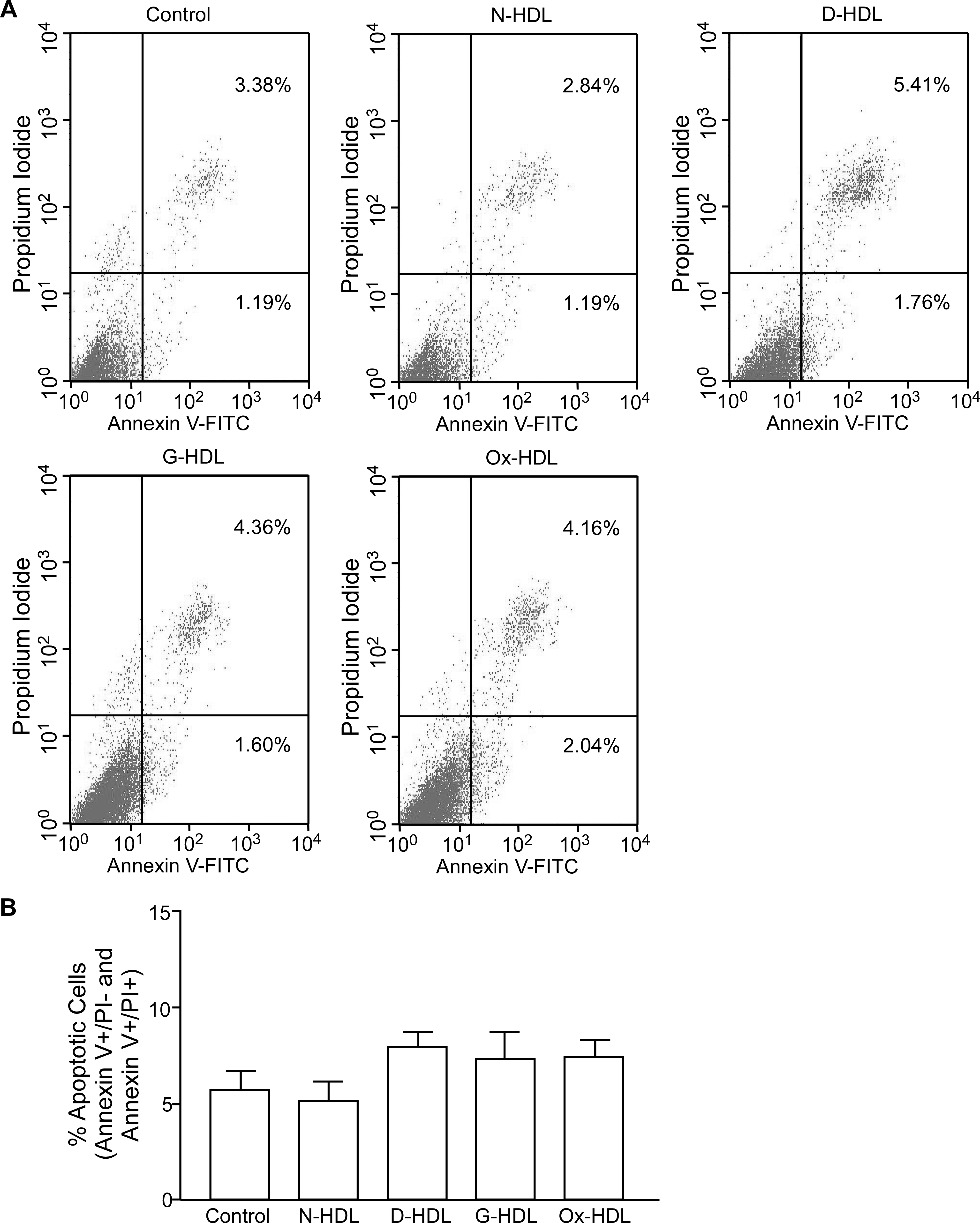

Supplement: Figure S1 — HUVEC apoptosis with normal, diabetic, glycated and oxidized HDL treatment. A. Flow cytometry histograms represent Annexin V-FITC staining in x axis and PI in y axis. The numbers represent the percentage of early (Annexin V+/PI−) [lower right quadrant] and late (Annexin V+/PI+) [upper right quadrant] apoptotic cells in HUVECs treated with PBS, N-HDL, D-HDL, G-HDL or Ox-HDL for 24 hours. B. There was no significant difference in the number of apoptotic cells between control cells and treated cells. N = 3, mean ± SEM, p>0.05 by ANOVA and Bonferroni’s Multiple Comparison Test. (DOC) [file pone.0048530.s001.doc]
